# Understanding the Challenges of HPV-Based Cervical Screening: Development and Validation of HPV Testing and Self-Sampling Attitudes and Beliefs Scales

**DOI:** 10.3390/curroncol30010093

**Published:** 2023-01-15

**Authors:** Ovidiu Tatar, Ben Haward, Patricia Zhu, Gabrielle Griffin-Mathieu, Samara Perez, Emily McBride, Aisha K. Lofters, Laurie W. Smith, Marie-Hélène Mayrand, Ellen M. Daley, Julia M. L. Brotherton, Gregory D. Zimet, Zeev Rosberger

**Affiliations:** 1 Lady Davis Institute for Medical Research (LDI), Jewish General Hospital, Montreal, QC H3T 1E2, Canada; 2 Research Center, Centre Hospitalier de l’Université de Montréal (CRCHUM), Montreal, QC H2X 0A9, Canada; 3 Cedars Cancer Centre, McGill University Health Centre (MUHC), Montreal, QC H4A 3J1, Canada; 4 Department of Oncology, McGill University, Montreal, QC H4A 3T2, Canada; 5 Institute of Psychiatry, Psychology & Neuroscience, King’s College London, London SE5 8AF, UK; 6 Department of Family and Community Medicine, University of Toronto, Toronto, ON M5G 1V7, Canada; 7 Department of Family and Community Medicine, Women’s College Hospital, Toronto, ON M5S 1B3, Canada; 8 BC Cancer Agency, Vancouver, BC V5Z 1G1, Canada; 9 Département d’Obstétrique-Gynécologie, Université de Montréal, Montreal, QC H3C 3J7, Canada; 10 College of Public Health, University of South Florida, Tampa, FL 33612, USA; 11 Melbourne School of Population and Global Health, University of Melbourne, Melbourne, VI 3010, Australia; 12 Population Health, Australian Centre for the Prevention of Cervical Cancer, Melbourne, VI 3053, Australia; 13 School of Medicine, Indiana University, Indianapolis, IN 46202, USA; 14 Department of Psychology, McGill University, Montreal, QC H3A 1G1, Canada

**Keywords:** cervical screening, HPV testing, HPV self-sampling, attitudes and beliefs, scale development, women’s health

## Abstract

The disrupted introduction of the HPV-based cervical screening program in several jurisdictions has demonstrated that the attitudes and beliefs of screening-eligible persons are critically implicated in the success of program implementation (including the use of self-sampling). As no up-to-date and validated measures exist measuring attitudes and beliefs towards HPV testing and self-sampling, this study aimed to develop and validate two scales measuring these factors. In October-November 2021, cervical screening-eligible Canadians participated in a web-based survey. In total, 44 items related to HPV testing and 13 items related to HPV self-sampling attitudes and beliefs were included in the survey. For both scales, the optimal number of factors was identified using Exploratory Factor Analysis (EFA) and parallel analysis. Item Response Theory (IRT) was applied within each factor to select items. Confirmatory Factor Analysis (CFA) was used to assess model fit. After data cleaning, 1027 responses were analyzed. The HPV Testing Attitudes and Beliefs Scale (HTABS) had four factors, and twenty-two items were retained after item reduction. The HPV Self-sampling Attitudes and Beliefs Scale (HSABS) had two factors and seven items were retained. CFA showed a good model fit for both final scales. The developed scales will be a valuable resource to examine attitudes and beliefs in anticipation of, and to evaluate, HPV test-based cervical screening.

## 1. Introduction

### Background

Cervical cancer is a threat to women and all persons with a cervix, and globally, it accounts for 311,000 deaths per year [1]. For multiple decades, the Papanicolaou (Pap) test was the primary method of screening to prevent cervical cancer. However, the Human papillomavirus (HPV) DNA test is now recognized as a superior method and recommended by numerous international health organizations [2,3,4,5] for primary cervical cancer screening (subsequently referred to as cervical screening) because of its higher sensitivity in detecting high-grade cervical lesions [6,7]. The HPV test is currently being used or is in implementation for cervical screening in several countries [8,9]. Primary HPV testing also provides opportunities for innovative approaches to cervical screening such as vaginal self-sampling, a promising approach to increase screening accessibility and uptake, particularly among those who are underscreened [10].

However, challenges to HPV primary screening implementation have been encountered by health authorities in several countries. In Australia and Wales, for example, there was inadequate population preparation and communication initially, which led to publicized resistance and petitions against the change to the HPV testing programs from long-standing cytology-based screening programs [11,12]. As other countries, including Canada, transition towards primary HPV testing as the new standard for cervical screening, it is critical to understand the psychosocial factors that underly HPV test acceptance and uptake to anticipate challenges to practice change.

Worldwide, cervical screening coverage falls significantly short of the World Health Organization’s elimination goals [13], and similarly, Canada has not met the targets set by the Canadian Partnership Against Cancer [14,15]. To increase acceptance and uptake of HPV testing, interventions need to consider not only how to increase knowledge, but also how to foster accurate beliefs and positive attitudes towards this screening method [16]. Findings from two systematic reviews suggest that acceptance of HPV testing varies across populations, and that perceived benefits of HPV-based screening, perceived susceptibility to HPV infection, and perceived subjective norms were facilitators of HPV testing acceptability [17,18]. Barriers to HPV testing acceptability included concerns about increased screening intervals and later ages of screening initiation associated with HPV primary screening policies, and negative emotions related to HPV testing (such as anxiety about test results and sexually transmitted infection-related stigma) [17,18]. These findings reinforce the need to identify concerns about the transition to HPV-based screening, and to design informative and reassuring communication strategies. In addition, self-sampling might present new issues, such as concerns about the accuracy of a self-collected test, the ability to follow-up in the case of a positive result, and the potential for injury [19,20,21,22]. At the same time, self-sampling could address many of the barriers of typical provider-administered screening, including cultural concerns (e.g., modesty and privacy), embarrassment, provider gender, and accessibility (e.g., finding and/or going to a clinic/doctor’s appointment or mobility challenges), while also providing greater patient autonomy [22,23,24,25].

Despite the critical importance of understanding attitudes and beliefs related to the HPV test and HPV self-sampling, there is a dearth of validated tools to measure these factors. Existing scales have been validated in specific populations that may not be representative [26] or have not undergone extensive psychometric testing [27]. There is a need for updated scales that are tested in a population-based sample and validated using advanced psychometric methods. The use of updated scales to accurately identify perceptions of HPV testing and self-sampling will in turn improve communication strategies from public health authorities related to HPV-based screening and help to pre-empt concerns that might disrupt changes to screening guidelines. Furthermore, validated HPV testing scales could reveal concerns relevant to specific higher-risk populations (e.g., underscreened) and highlight opportunities to increase cervical screening uptake. Therefore, the aim of the current study was to develop and validate two scales: one measuring attitudes and beliefs towards HPV testing, and a second measuring attitudes and beliefs towards self-sampling.

## 2. Materials and Methods

### 2.1. Participants and Study Design

A web-based survey was administered from October to November 2021. Inclusion criteria were being biologically female, living in Canada, and being aged 21 to 70. Exclusion criteria were not having a cervix (e.g., due to hysterectomy) or having a previous diagnosis of cervical cancer. Census-based quotas were applied for primary language (English or French) and province or territory of residence. Oversampling was used to ensure that approximately half of the sample were underscreened for cervical cancer (>3 years since previous screening with the Pap test-based on Canadian screening recommendations) to ensure the validity of the developed scales in underscreened populations. Participants were recruited by Dynata, a market research firm with a large panel of Canadian residents who were invited to complete a survey about “Health and Wellness”.

The study was conducted as part of a pilot project in preparation for a larger survey investigating the psychosocial and sociodemographic correlates of HPV test intentions among screening-eligible Canadians. A detailed description of the study’s methodology can be found elsewhere [28]. Participants completed an online questionnaire that took approximately 25 min to complete and included items pertaining to socio-demographics, health behaviours, HPV testing-related knowledge (see published knowledge scales [29]), and attitudes and beliefs. The present study is focused on the HPV testing attitudes and beliefs items. At the time of the survey, HPV-based cervical screening had not been implemented in Canada. Therefore, participants were presented with several informative statements throughout the questionnaire which provided minimal information necessary for participants to understand the context of the survey questions. The study received ethical approval from the Research Ethics Board of The Integrated Health and Social Services University Network (CIUSSS) West-Central Montreal (Project ID: 2021-2632).

### 2.2. Measures

To identify items to include in the survey, an extensive literature review of existing measures was conducted which identified 13 relevant scales. A pool of 781 items was then created from these existing measures, as well as from items and themes identified in the literature [18,30]. Informed by the results of a systematic review conducted by our team [18] that used the Health Belief Model (HBM) and Theory of Planned Behavior (TBP) [31,32] to map factors associated with HPV test acceptability, these items were then categorized into two potential scales relating to attitudes and beliefs about HPV testing and self-sampling. Of these items, 369 were related to HPV testing attitudes and beliefs, and 184 to HPV self-sampling attitudes and beliefs; the rest of the items were used to develop the cervical cancer and HPV testing knowledge scales, and results have been published elsewhere [29]. All potential attitude and beliefs items were reviewed by the research team (ZR, OT, GGM, PZ, SP) and selected by consensus for inclusion in the survey. Selection criteria included applicability to current or proposed screening guidelines and significant findings from the extant literature. Items were then refined in consultation with both national and international researchers involved in cervical screening programs and cervical cancer prevention. Items were translated into French by a professional translation service. To ensure comprehension and clarity, items were tested and revised in cognitive interviews in both English and French, with seven Canadians who met the study’s inclusion criteria. Participants were recruited using advertisements placed on relevant Canada-based social media groups (e.g., “Montreal Moms”, “McGill Psychology Students Association”).

In total, 44 items related to HPV testing attitudes and beliefs and 13 items related to HPV self-sampling attitudes and beliefs were retained for inclusion in the questionnaire. In each section, we added one attention check item to facilitate the identification of “inattentive” responses during data cleaning. These items can be found in Supplementary Material File S1: All Included Items. All attitudes and beliefs items were designed to be answered using a seven-point Likert scale: (1) *strongly disagree*, (2) *disagree*, (3) *somewhat disagree*, (4) *neutral*, (5) *somewhat agree*, (6) *agree*, and (7) *strongly agree*. A more detailed overview of the measures (including sociodemographic variables) used in this study is described in the study protocol [28].

An Informative statement was presented before each of the HPV testing and HPV self-sampling attitudes and beliefs sections that highlighted the differences between the HPV test and Papanicolaou (Pap) test and showed the procedure to conduct self-sampling, respectively. These informative statements are available in Supplementary Material File S2: All Informative Statements.

### 2.3. Statistical Analyses

From the final dataset (*N* = 1027), we randomly selected about half of the observations (*n* = 512) for exploratory factor analyses (EFA) and Item Response Theory (IRT) modelling and performed confirmatory factor analyses (CFA) on the other half of the dataset (*n* = 515). On the first dataset (*n* = 512), we used EFA with maximum likelihood extraction and oblimin rotation that accounts for inter-factor correlation to explore factor structure. To evaluate the optimal number of factors to be extracted in EFA, we used the parallel analysis syntax developed by O’Connor [33] and retained factors with higher Eigenvalues in the actual dataset than randomly simulated Eigenvalues. For within-factor items, we used IRT and graded response models for categorical data. We evaluated item discrimination (i.e., variation in the response probability as a function of latent construct ability levels) and information curves slopes (higher slopes reflect higher information value) obtained by plotting item information against the latent construct ability (theta). We retained items with higher discrimination and information, and items that provided information at extreme theta values. For items with similar information and discrimination, we examined the category characteristic curves that show the probability of selecting Likert scale ratings as a function of theta values, and flagged items with low response variability (e.g., most likely to choose “neither agree nor disagree” over a large theta range). The iterative process of selecting items also included examining their conceptual value and changes in the shape of the test information function (which reflects the summative information value of items included in a factor) after eliminating flagged items.

Using the second dataset (*n* = 515), we performed confirmatory factor analyses (CFA) and allowed for within-factor correlation of error terms as suggested by modification indices. To provide a comprehensive picture of model fit, the following indices were reported: (a) Wheaton et al.’s relative/normed chi-square (χ^2^/df, recommended values 2 to 5), (b) the standardized root mean square residual (SRMR), (c) the root mean square error approximation (RMSEA), (d) the comparative fit index (CFI), and (e) the Tucker-Lewis index (TLI) [34]. Based on the recommendations of Hu and Bentler [35], three index combinations (and cut-off criteria) can be used to evaluate if a model fits well: (a) TLI of 0.96 or higher and an SRMR of 0.9 or lower; (b) RMSEA of 0.06 or lower and a SRMR of 0.09 or lower; or (c) CFI of 0.96 or higher and a SRMR of 0.09 or lower. We performed subgroup CFA analyses based on screening status (adequate and inadequate screening participation) and language (English and French). The reliability of each subscale was calculated using Cronbach’s α and McDonald’s ω. In contrast with Cronbach’s α, McDonald’s ω does not assume equal factor loadings, a condition that is rarely met in scale development [36,37]. Each subscale was named according to conceptual similarities of the final retained items. To examine criterion validity, we used independent samples t-tests to compare the mean score for each sub-scale between adequately screened and underscreened participants, and between participants who intended and those who did not intend to use the HPV test or self-sampling for screening. Effect sizes were calculated using Cohen’s d. Statistical analyses were performed using STATA 17.0 [38] and IBM SPSS v. 24 [39].

## 3. Results

In total, 1230 participants completed the survey and, after data cleaning methods were applied to identify potentially inattentive or unmotivated respondents [40], 203 responses were excluded (see protocol paper [28] for a detailed overview of data cleaning methods). Of the remaining 1027 respondents, 503 reported being adequately screened for cervical cancer, and 524 reported being underscreened. See Table 1 for an overview of the sociodemographic characteristics of the sample.

### 3.1. HPV Testing Attitudes and Beliefs

EFA, using all 44 items included in the survey, revealed 11 factors with Eigenvalues greater than 1 and parallel analyses showed that from a statistical standpoint, the maximum number of factors extracted should not be higher than five. We compared in EFA the five-factor and a more parsimonious four-factor solution and decided that the latter provided a better solution because the fifth extracted factor (four items) included two items with cross-loadings higher than 0.6 (items 30 and 31), and one item with a low EFA loading (0.24; item 27). Our decision aligned with the results of the log-likelihood test showing a lower Bayesian Information Criterion value for the four-factor than for the five-factor solution (77,307 and 77,335, respectively) (see Supplementary Material File S3: Exploratory Factor Analyses, Table A in Supplementary Material File S3 for results of parallel analysis and Table B in Supplementary Material File S3 for EFA loadings).

For items loading on factor 1, and using IRT analyses, we decided to remove items 33, 4, 21, and 24 as these had the lowest discrimination and information (See Supplementary Material File S4: Item Response Theory Analyses, Table A in Supplementary Material File S4). Items 20 and 8 showed similar discrimination and information, and we retained item 8 because of higher information at lower theta values. Item 7 and 11 showed low information value and response variability and were removed. In factor 2, items 22, 6, 9, and 10 were removed based on low information and discrimination. We removed 9 out of 18 items extracted in factor 3 based on their low information value across all values of the latent variable: 30, 14, 39, 15, 36, 25, 13, 23, 27. We decided to remove item 17 “…the HPV test would be a good way to detect early abnormal changes in the cervix” because of the similar face validity with item 16 “…having the HPV test would be a good way to identify problems before they become cancer” which had higher discriminant and information than item 17. Items 31 and 45 had similar information, and we kept item 45 because of better discrimination. Items 35 and 43 performed almost identically in terms of information and discrimination, and we kept item 43 because we considered that measuring perceptions about healthcare professionals’ opinion is of higher relevance for a scale that could be used independent of the existence of organized cervical screening programs. Removing 12 items only slightly changed the shape of the test information function, as shown in Figure G in Supplementary Material File S5. In factor 4, item 29 was removed based on very low information. Item 32 was retained because it provided information at lower and higher values of the latent variable and showed acceptable discrimination.

On the second dataset (*n* = 515), we conducted CFA analyses for the HPV attitudes and beliefs scale that consists of 20 items grouped in four scales that were named *Personal Barriers* (7 items); *Social Norms* (4 items); *Confidence* (6 items); and *Worries* (3 items) (See Table 2). The scales showed good to very good reliability, as shown by (Cronbach’s α; McDonald’s ω) values of 0.815; 0.818 for *Personal Barriers*, 0.787; 0.790 for *Social Norms*, 0.780; 0.774 for *Confidence*, and 0.654; 0.673 for *Worries*. Results of the goodness of fit tests confirmed good model fit based on RMSEA and SRMR in the second dataset and in all subgroups (See Table 3). Criterion validity analyses found significant differences on *Personal Barriers* and *Confidence* between those who were underscreened and adequately screened and those who did and did not intend to use the HPV test, and a significant difference on the *Worries* factor between those who did and did not intend to use the HPV test. No significant differences were observed based on screening status or HPV test intentions for the *Social Norms* factor. The full results of the criterion validity analyses for the HPV Testing Attitudes and Beliefs Scale (HTABS) are shown in Table 4.

### 3.2. HPV Self-Sampling Attitudes and Beliefs

Initial EFA analyses using all 13 items included in the survey revealed three factors with Eigenvalues greater than 1, but we extracted two factors based on results of parallel analysis (See Tables C and D in Supplementary Material File S3). Pertaining to items loading on factor 1, we removed items 6, 14, and 5 because of low information values (See Table B in Supplementary Material File S4). Item 9 “…HPV self-sampling would be easy to do” was not included in the final scale because of modest information and high cross-loading (0.7) in EFA. Items 1 and 8, corresponding to the second factor, were eliminated because of low information across all theta values.

The final self-sampling scale included a total of seven items distributed in two scales: *Concerns* (4 items) and *Autonomy* (3 items) (See Table 5 for item analyses on the second dataset). The two scales had very good reliability based on selected indices (Cronbach’s α; McDonald’s ω) of 0.769; 0.779 for *Concerns* and 0.822; 0.829 for *Autonomy*. The scale had adequate fit on the second dataset and in all four subgroups, as most fit indices exceeded cut-off criteria (See Table 6). See Supplementary Material File S5 for the graphical representation of the item information functions, and the corresponding test characteristic curves using the full sample (*N* = 1027) for all subscales. On the *Autonomy* factor, a significant difference in scores was observed between adequately screened and underscreened participants, and those who did and did not intend to use self-sampling for screening. A significant difference was only observed between those who did and did not intend to use self-sampling for the *Concerns* factor. The full results of the criterion validity analyses for the HPV Self-Sampling Attitudes and Beliefs Scale (HSABS) are available in Table 7.

## 4. Discussion

In this study, we aimed to develop and validate two scales measuring attitudes and beliefs regarding HPV testing and self-sampling to better understand how these factors might influence the acceptance of HPV-based primary screening programs. The resulting scales measure multiple dimensions of HPV test-related attitudes, were tested in both English and French, and demonstrated robust psychometric properties. In addition, validation in a national sample of screening-eligible Canadians, with oversampling of underscreened participants, should encourage the use of these scales in populations that require specific attention to address deficits in screening acceptability and uptake.

The final HPV Testing Attitudes and Beliefs (HTABS) scale contains 20 items loading onto four factors (see Supplementary Material File S6: Final HPV Attitudes and Beliefs Scale and HPV Self-Sampling Attitudes and Beliefs Scale for final scales and item numbers). The first factor of the HTABS, *Personal Barriers*, includes items related to stigma and embarrassment related to screening and to HPV as a sexually transmitted infection, the personal priority of screening, inconvenience, exam-specific fears, and beliefs that testing is only needed when symptoms are present. It is conceptually similar to other subscales identified in existing measures of cervical screening beliefs, which implicate these as key factors in screening engagement [41,42,43]. The *Social Norms* factor was similar to the “Subjective Norms: Indirect” subscale developed by Ogilvie et al., [27] in considering the importance of friends and partners’ opinions towards HPV test-based screening; although, it included additional items that were related to the influence of opinions from family and social media. In contrast with the results of the study published by Ogilvie et al., [27], we found that items asking about the need for opinions from physicians and health authorities loaded onto the third factor, *Confidence*, rather than *Social Norms*. Our results could suggest that while the opinions of health authorities and physicians are technically the opinions of others, these opinions might be considered as an objective indication of confidence in the HPV test and procedure, while the opinions of family, friends, and social media provide a normative indication of what ‘ought’ to be done. The *Confidence* factor also enveloped items about the perceived benefit and safety of HPV testing, which are critical components of screening acceptability [17,18]. The fifth and final factor, *Worries*, included items involving concerns about increases to screening intervals and ages of initial screening, which have been notable contentions to HPV primary screening implementation [12,44], and a subject of concern for those eligible for screening and healthcare professionals [17,45,46,47,48,49]. A recent study conducted by our research team suggested that both adequately screened and underscreened participants preferred shorter screening intervals and younger ages of screening, regardless of testing method, which may be driven by cancer-related worry [50].

In total, 24 items were excluded from the final model. Notably, items related to worries about transmission to a partner (Item 23), unfaithfulness of a partner (Item 24), and communication with a partner (Item 36) were excluded, despite being reported as reactions to positive HPV test results [30,51]. Our findings might suggest that these are more indicative of interpersonal HPV or STI-related attitudes and beliefs rather than HPV test-specific concerns. Criterion validity analyses for the HTABS revealed adequately screened participants had lower scores on the *Personal Barriers* and higher scores on the *Confidence* subscales. Similar results for those who intended to screen with the HPV test versus those who did not intend to screen with it suggests that these two factors are useful in identifying barriers to HPV-based screening and provide meaningful insight into attitudes toward the test and procedure effectiveness that might predict acceptability. Interestingly, a significant difference in the *Worries* subscale was observed between the two HPV test intention groups and indicates that those who intend to use the test had higher worries regarding proposed changes to screening intervals and ages. It is possible that those who do not intend to use the HPV test for screening are not concerned about the implications of changes to screening intervals and ages considering they are disengaged with the screening method itself, while those who do intend to use the HPV test, despite being confident in the test itself, are more likely to consider these factors.

After item reduction, the final HPV Self-Sampling Attitudes and Beliefs Scale (HSABS) contained seven items loading onto two factors (see Supplementary Material File S6 for final scales and item numbers). To our knowledge, it is the only scale measuring self-sampling-specific attitudes and beliefs that has been validated in a national sample. This scale expands on and updates the scale developed by Kahn et al. [26], which was tested in a sample of adolescents who would likely not be offered routine HPV-based screening under current guidance [2,5]. The *Concerns* factor included an item pertaining to confidence in using a self-sampling kit and the potential for harm with self-sampling, which were both commonly reported components of self-sampling acceptability identified in systematic reviews [19,20]. In addition, the item “I would feel embarrassed doing HPV self-sampling” addresses the perceived embarrassment of self-sampling, the reduction of which is noted as a key facilitator of acceptability versus clinician-administered sampling [19,20]. The *Autonomy* factor included items related to preferences in comfort and travelling between self and clinician-administered sampling, both of which have been noted as reasons to accept self-sampling, particularly for underscreened and otherwise hard-to-reach populations [52,53,54,55]. A final item asked participants to rate their feeling of body self-determination using self-sampling. A synthesis of qualitative studies by Camara et al. [22] suggested that while for some cultural factors invoking “body shyness” were a deterrent to self-sampling, other studies found that self-sampling was seen to provide an opportunity for participants to build comfort and experience empowerment with their body. Notably, a study by McDowell et al. [56] found that trans-masculine individuals, a group facing significant structural and psychosocial barriers to cervical screening [57], felt a greater sense of agency from self-sampling and preferred it to clinician-administered sampling.

Those who intended to use HPV Self-Sampling demonstrated lower scores on *Concerns* compared to those who did not intend to use the test, confirming this subscale’s value in predicting acceptability and uptake of self-sampling. Significantly higher scores on the *Autonomy* subscale in underscreened compared to adequately screened participants align well with existing findings, demonstrating that self-sampling is highly acceptable and advantageous to increase uptake in this group [10,50]. In addition, higher scores on this scale among those who intended to use HPV self-sampling suggest it predicts intentions to use this method for screening and would be useful for health authorities to confirm the utility of adding self-sampling as an option in screening programs.

### Limitations

The study has several limitations that could be addressed in further applications of the developed scales. While the scales were developed in a large national sample of screening-eligible Canadians, and validated in both English and French, future studies in different cultural contexts and populations could confirm its wider applicability. Furthermore, certain items, such as those in the *Worries* subscale of the HTABS relating to specific changes to screening intervals and screening ages, which are applicable to Canada and aligned with most screening recommendations, might not be relevant in lower-and-middle income countries with structural and resource constraints that prevent regular screening [58]. The present study was conducted as part of a larger investigation of psychosocial factors impacting HPV test intentions [28] and scales such as those produced by Ogilvie et al. [27] and Kahn et al. [26] were not included in the survey, preventing the examination of convergent, divergent, and concurrent validity with existing measures. The study was cross-sectional, precluding the examination of test-retest reliability.

## 5. Conclusions

The introduction of HPV-based screening programs worldwide provides an opportunity to significantly reduce morbidity and mortality from cervical cancer. However, understanding and addressing negative perceptions towards HPV testing and associated policy changes is crucial to maintaining engagement with, and trust in, screening programs. In addition, examining attitudes and beliefs about self-sampling could inform targeted messaging strategies highlighting benefits and addressing concerns related to this novel screening approach, especially in inadequately screened populations. The developed scales, which draw upon findings in the extant literature, are informed by theoretical frameworks, were validated in English and French, and are valuable tools for future investigations and to standardize measurement across studies.

## Figures and Tables

**Table 1:** Sample Characteristics (*N* = 1027).

Variable	Total (*N* = 1027)	Adequately Screened (*n* = 503)	Underscreened (*n* = 524)	Between-Group Difference ^a^ *p* Value
Age (yr), mean (SD)	48.36 (12.58)	48.80 (12.02)	47.94 (13.08)	0.28
Gender, *n* (%)				
Female	1023 (99.6)	501 (99.6)	522 (99.6)	0.51
Other	4 (0.4)	2 (0.4)	2 (0.4)	
Ethnicity ^b^, *n* (%)				
North American Aboriginal	30 (3.0)	17 (3.4)	13 (2.5)	0.02
Other North American	461 (44.9)	231 (45.9)	230 (43.9)	
European	340 (33.1)	176 (35.0)	164 (31.3)	
Asian	139 (13.5)	50 (9.9)	89 (17.0)	
Other	57 (5.5)	29 (6.7)	28 (5.3)	
Self-perceived visible minority, *n* (%)				
Yes	195 (19.0)	120 (22.9)	75 (14.9)	<0.01
No	832 (81.0)	383 (77.1)	449 (85.1)	
Canadian region, *n* (%)				
Western	313 (30.5)	157 (31.2)	156 (29.8)	0.01
Central	651 (63.4)	303 (60.2)	348 (66.4)	
Eastern	61 (5.9)	42 (8.3)	19 (3.6)	
Territories	2 (0.2)	1 (0.3)	1 (0.2)	
Primary language spoken at home, *n* (%)				
English	765 (74.5)	394 (78.3)	371 (70.8)	0.02
French	211 (20.5)	90 (17.9)	121 (23.1)	
Other	51 (5.0)	19 (3.8)	32 (6.1)	
Any post-secondary education ^c^, *n* (%)				
Yes	718 (69.9)	359 (71.4)	359 (68.5)	0.32
No	309 (30.1)	144 (28.6)	165 (31.5)	
Employment status, *n* (%)				
Employed full-time	496 (48.3)	273 (54.3)	223 (42.6)	<0.001
Employed part-time	131(12.7)	58 (11.5)	73 (13.9)	
Not employed	95 (9.3)	33 (6.6)	62 (11.8)	
Student	16 (1.6)	4 (0.8)	12 (2.3)	
Retired	182 (17.7)	84 (16.7)	98 (18.7)	
Caregiver	58 (5.6)	33 (6.6)	25 (4.8)	
Other	49 (4.8)	18 (3.6)	31(5.9)	
Household income, *n* (%)				
Below $60,000	454 (44.2)	182 (36.2)	272 (51.9)	<0.001
Above $60,000	554 (53.9)	312 (62.0)	242 (46.2)	
Prefer not to answer	19 (1.9)	9 (1.8)	10 (1.9)	
Living in Canada for past 10 years or more, *n* (%)				
Yes	990 (96.4)	490 (97.4)	500 (95.4)	0.09
No	37 (3.6)	13 (2.6)	24 (4.6)	
Relationship status, *n* (%)				
Married/common law partner	611 (59.5)	326 (64.8)	285 (54.4)	<0.01
Single	377 (36.7)	155 (30.8)	222 (42.4)	
Dating	39 (3.8)	22 (4.4)	17 (3.2)	

Note: ^a^ Between-group analyses for adequately and underscreened participants were conducted using independent samples t-tests for continuous data and Pearson’s chi-squared test for categorical data. ^b^ Includes Caribbean, Latin, Central and South American, African, Oceania, and other (i.e., incomprehensible or “mixed” responses provided in free response). ^c^ Includes any apprenticeship, trade certificate/diploma, and/or college or university degree.

**Table 2:** Results of factor analyses and IRT analyses for the final items of the HPV Testing Attitudes and Beliefs Scale items (20 items).

Item	EFA (*n* = 515)	Discrimination (*n* = 515)	Information (*n* = 515)	CFA (*n* = 515)
I feel that …				
Personal barriers				
2. … going to see a healthcare professional to have the HPV test would take too much time	0.727	2.53	2.00	0.64
1. … I would be embarrassed to show my genitals to a healthcare professional during the HPV test	0.691	1.74	0.97	0.48
5. … I have other priorities more important than having the HPV test	0.583	1.55	0.75	0.43
8. … the HPV test would be painful	0.567	1.18	0.44	0.44
19. … I would be embarrassed to get tested for HPV because it is a sexually transmitted infection	0.526	1.64	0.85	0.70
12. … I would not need to have the HPV test because I do not have symptoms	0.413	1.71	0.91	0.72
3. … healthcare professionals doing the HPV test would be rude to me	0.413	1.71	0.92	0.82
Social norms				
40. … my friends’ opinion about getting the HPV test would be important to me	0.752	3.21	3.20	0.81
41. … my family’s opinion about getting the HPV test would be important to me	0.720	2.56	2.07	0.64
42. … my partner’s opinion about getting the HPV test would be important to me	0.634	1.61	0.82	0.49
44. … opinions I see on social media (for example, on Facebook, Twitter, Instagram, etc.) about getting the HPV test would be important to me	0.563	1.76	0.97	0.67
Confidence				
16. … having the HPV test would be a good way to identify problems before they become cancer	0.762	3.01	2.72	0.65
26. … if the HPV test showed I have HPV, it is important to follow up on it	0.725	2.42	1.83	0.57
34. … if I learn that I have an HPV infection, I feel that I would need more information to help me deal with the results	0.647	1.74	0.95	0.37
43. … my healthcare professional’s opinion about getting the HPV test would be important to me	0.485	1.45	0.67	0.43
18. … the HPV test would be safe	0.439	1.54	0.72	0.74
45. … public health agencies’ opinions about getting the HPV test would be important to me	0.406	1.07	0.36	0.34
Worries				
38. … I would be worried about starting screening for cervical cancer with the HPV test at 30 years old instead of 21 years old	−0.659	1.77	0.98	0.49
37. … I would be worried about starting screening for cervical cancer with the HPV test at 25 years old instead of 21 years old	−0.631	1.88	1.10	0.78
32. … I would be worried about getting tested with the HPV test less often than every 3 years	−0.527	1.29	0.52	0.70

Note: EFA denotes Exploratory Factor analysis with maximum likelihood extraction and oblimin rotation for a 4-factor solution; In the information column are provided maximum information values as per item information functions. CFA denotes Confirmatory Factor Analysis. The CFA column contains standardized regression coefficients. Total variance explained by the 4-factor solution = 54.99%.

**Table 3:** CFA model fit indices for the HPV Testing Attitudes and Beliefs Scale (20 items).

Fit Indices	First Dataset (*n* = 512)	Second Dataset (*n* = 515)	Adequately Screened (*n* = 503)	Not Adequately Screened (*n* = 524)	English (*n* = 820)	French (*n* = 207)
Wheaton’s χ^2^/df	**2.56**	**2.43**	1.90	**3.03**	**3.30**	1.59
SRMR	**0.06**	**0.06**	**0.05**	**0.07**	**0.06**	**0.07**
RMSEA	**0.06**	**0.05**	**0.04**	**0.06**	**0.05**	**0.05**
CFI	0.94	0.94	**0.96**	0.92	0.94	0.94
TLI	0.91	0.91	0.94	0.87	0.91	0.92

Note: Fit indices that met cut-off criteria are bolded.

**Table 4:** Criterion validity analyses for the HPV Testing Attitudes and Beliefs Scale.

Subscales	Full Sample M (SD)	Adequately Screened M (SD)	Under Screened M (SD)	*p*	Cohen’s d	HPV Test Intenders M (SD)	HPV Test Non-Intenders M (SD)	*p*	Cohen’s d
Personal Barriers	3.06 (1.11)	2.76 (1.06)	3.35 (1.07)	<0.001	−0.55	2.71 (1.12)	3.21 (1.06)	<0.001	−0.46
Social Norms	3.14 (1.36)	3.13 (1.38)	3.16 (1.34)	0.753	−0.02	3.06 (1.45)	3.18 (1.32)	0.196	−0.09
Confidence	5.62 (0.82)	5.78 (0.78)	5.46 (0.83)	<0.001	0.39	5.85 (0.74)	5.52 (0.83)	<0.001	0.41
Worries	3.77 (1.19)	3.81 (1.19)	3.73 (1.18)	0.256	0.07	3.99 (1.28)	3.68 (1.13)	<0.001	0.26

**Table 5:** Results of factor (EFA; CFA) and IRT analyses for the final items of the HPV Self-Sampling Attitudes and Beliefs Scale items (7 items).

Item	EFA (*n* = 515)	Discrim. (*n* = 515)	Inform. (*n* = 515)	CFA (*n* = 515)
I feel that …				
Concerns				
2. … if I did HPV self-sampling, I would worry that I am not doing it right	0.417	1.10	0.39	0.77
3. … if I did HPV self-sampling, I could harm myself	0.889	4.34	5.50	0.60
4. … if I did HPV self-sampling, I could get an infection	0.818	2.67	2.21	0.39
7. … I would feel embarrassed doing HPV self-sampling	0.573	1.72	0.95	0.65
Autonomy				
10. … I would be more comfortable doing the swab by myself using HPV self-sampling than having an HPV test done by a healthcare professional	0.803	2.69	2.25	0.71
12. … I would prefer doing HPV self-sampling at home because it would save me travelling to see a healthcare professional	0.787	2.74	2.36	0.88
13. … if I did HPV self-sampling, I would be more in control of my body	0.771	2.67	2.20	0.76

Note: EFA denotes Exploratory Factor analysis with maximum likelihood extraction and oblimin rotation for a 2-factor solution; In the information column are provided maximum information values as per item information functions; CFA denotes Confirmatory Factor Analysis. The CFA column contains standardized regression coefficients. Total variance explained by the 2-factor scale = 67.19%.

**Table 6:** CFA model fit indices for the HPV Self-Sampling Attitudes and Beliefs Scale (7 items).

Fit Indices	First Dataset (*n* = 512)	Second Dataset (*n* = 515)	Adequately Screened (*n* = 503)	Not Adequately Screened (*n* = 524)	English (*n* = 820)	French (*n* = 207)
Wheaton’s χ^2^/df	**3.72**	1.68	**2.87**	**2.66**	**4.21**	**2.40**
SRMR	**0.02**	**0.01**	**0.03**	**0.02**	**0.02**	**0.06**
RMSEA	0.07	**0.04**	**0.06**	**0.06**	**0.06**	0.08
CFI	**0.99**	**1.00**	**0.99**	**0.99**	**0.99**	**0.97**
TLI	**0.96**	**0.99**	**0.97**	**0.97**	**0.97**	**0.95**

Note: Fit indices that met cut-off criteria are bolded.

**Table 7:** Criterion validity analyses for the HPV Self-Sampling Attitudes and Beliefs Scale.

Subscales	Full Sample M (SD)	Adequately Screened M (SD)	Under Screened M (SD)	*p*	Cohen’s d	HPV Self-Sampling Intenders M (SD)	HPV Self-Sampling Non-Intenders M (SD)	*p*	Cohen’s d
Concerns	3.15 (1.21)	3.19 (1.23)	3.11 (1.19)	0.302	0.06	2.65 (1.12)	3.38 (1.18)	<0.001	−0.63
Autonomy	4.75 (1.41)	4.45 (1.45)	5.04 (1.31)	<0.001	−0.43	5.40 (1.22)	4.45 (1.39)	<0.001	0.71

## Data Availability

The data sets used for this study will not be published in a publicly available repository, in accordance with the ethics proposal approved by the overseeing research ethics board. They will be available from the senior author (ZR) on reasonable request, and upon agreement of confidentiality and data use policies provisioned by the primary institution.

## Supplementary Materials

The following supporting information can be downloaded at: https://www.mdpi.com/article/10.3390/curroncol30010093/s1, Supplementary Material File S1: All Included Items; Supplementary Material File S2: Informative Statements; Supplementary Material File S3: Exploratory Factor Analyses; Supplementary Material File S4: Item Response Theory Analyses; Supplementary Material File S5: Item Information Functions and Test Characteristic Curves; Supplementary Material File S6: Final HPV Attitudes and Beliefs Scale and HPV Self-Sampling Attitudes and Beliefs Scale.
