# Supplementary material for: Understanding the Challenges of HPV-Based Cervical Screening: Development and Validation of HPV Testing and Self-Sampling Attitudes and Beliefs Scales
Source: Curr Oncol. 2023 Jan 15;30(1):1206–19. doi: 10.3390/curroncol30010093 (PMC9858121; doi:10.3390/curroncol30010093)
Supplement: Supplementary file 1 [file curroncol-30-00093-s001.zip › Flie S1 All Included Items.pdf]

Supplementary Material File S1.

All Included Items

Attitudes and Beliefs about HPV Testing

The following sections will present a series of statements about HPV testing. Please note that we are not asking about your knowledge. Whether or not you have had an HPV test in the past, we wish to know about your attitudes and beliefs. If you are unsure how to answer any question, simply select the answer that best reflects your attitudes and beliefs.

Statement	Strongly disagree (1)	Disagree (2)	Somewhat disagree (3)	Neutral (4)	Somewhat agree (5)	Agree (6)	Strongly agree (7)
I feel that …							
1. … I would be embarrassed to show my genitals to a healthcare professional during the HPV test							
2. … going to see a healthcare professional to have the HPV test would take too much time							
3. … healthcare professionals doing the HPV test would be rude to me							
4. … that I would not remember to have the HPV test regularly							
5. … I have other priorities more important than having the HPV test							
6. … if the HPV test showed I have HPV, it would mean I have cancer							
7. … I would be uncomfortable with a male health care professional doing my HPV test							
8. … the HPV test would be painful							

Statement	Strongly disagree (1)	Disagree (2)	Somewhat disagree (3)	Neutral (4)	Somewhat agree (5)	Agree (6)	Strongly agree (7)
I feel that …							
9. … I would need the HPV test because I am at risk of developing cervical cancer							
10. … having the HPV test would prevent me from getting cervical cancer							
11. … I would not need to have the HPV test because I only have one sexual partner							
12. … I would not need to have the HPV test because I do not have symptoms							
13. … it would be simple for me to have access to a healthcare professional to receive the HPV test							
14. … having the HPV test would be entirely up to me							
15. … I do not want to have the HPV test because it would affect my sex life							
16. … having the HPV test would be a good way to identify problems before they become cancer							
17. … the HPV test would be a good way to detect early abnormal changes in the cervix							
18. … the HPV test would be safe							

Statement	Strongly disagree (1)	Disagree (2)	Somewhat disagree (3)	Neutral (4)	Somewhat agree (5)	Agree (6)	Strongly agree (7)
I feel that …							
19. … I would be embarrassed to get tested for HPV because it is a sexually transmitted infection							
20. … if the HPV test showed I have HPV, I would be ashamed							
21. … I would not trust the HPV test as much as the Pap test for my routine cervical cancer screening							
22. … if the HPV test showed I have HPV, I would be worried about my ability to have children							
23. … if the HPV test showed I have HPV, I would worry about giving my partner HPV							
24. … if the HPV test showed I have HPV, I would suspect my partner of being unfaithful							
25. … if the HPV test showed I have HPV, I would not really be worried							
26. … if the HPV test showed I have HPV, it is important to follow up on it							
27. … if the HPV test showed I have HPV, this result should not be delivered over the phone							

Statement	Strongly disagree (1)	Disagree (2)	Somewhat disagree (3)	Neutral (4)	Somewhat agree (5)	Agree (6)	Strongly agree (7)
I feel that …							
28. … to show us that you are reading the questions carefully, please select “strongly agree”, for this question only. [ATTENTION CHECK ITEM]*							
29. … if the government increases the time between screenings to more than 3 years, this would be done solely to reduce costs							
30. … getting more information about the HPV test would reduce my fear about this test							
31. … getting more information about the HPV test may reduce my fear about increasing time between screenings to more than 3 years							
32. … I would be worried about getting tested with the HPV test less often than every 3 years							
33. … I do not have enough information to make a decision about getting screened with the HPV test							
34. … if I learn that I have an HPV infection, I feel that I would need more information to help me deal with the results							

Statement	Strongly disagree (1)	Disagree (2)	Somewhat disagree (3)	Neutral (4)	Somewhat agree (5)	Agree (6)	Strongly agree (7)
I feel that …							
35. … it would be important to get a reminder to go for my cervical cancer screening (for example, with a mailed reminder letter)							
36. … if the HPV test showed I have HPV, I would be comfortable sharing this information with my partner							
37. … I would be worried about starting screening for cervical cancer with the HPV test at 25 years old instead of 21 years old							
38. … I would be worried about starting screening for cervical cancer with the HPV test at 30 years old instead of 21 years old							
39. … if the HPV test showed I have HPV, it would be serious							
40. … my friends' opinion about getting the HPV test would be important to me							
41. … my family's opinion about getting the HPV test would be important to me							
42. … my partner's opinion about getting the HPV test would be important to me							
43. … my healthcare professional's opinion about getting the HPV test would be important to me							

Statement	Strongly disagree (1)	Disagree (2)	Somewhat disagree (3)	Neutral (4)	Somewhat agree (5)	Agree (6)	Strongly agree (7)
I feel that …							
44. … opinions I see on social media (for example, on Facebook, Twitter, Instagram, etc.) about getting the HPV test would be important to me							
45. … public health agencies' opinions about getting the HPV test would be important to me							

Note: * denotes attention check items used for data cleaning that were not retained in CFA and EFA analyses

Attitudes and Beliefs about HPV Self-sampling

The following sections will present a series of statements about HPV self-sampling. Please note that we are not asking about your knowledge. Whether or not you have done HPV self-sampling in the past, we wish to know your attitudes and beliefs. If you are unsure how to answer any question, simply select the answer that best reflects your attitudes and beliefs.

Statement	Strongly disagree (1)	Disagree (2)	Somewhat disagree (3)	Neutral (4)	Somewhat agree (5)	Agree (6)	Strongly agree (7)
I feel that …							
1. … I would prefer doing HPV self-sampling because it would avoid my family knowing that I am being screened for cervical cancer							
2. … if I did HPV self-sampling, I would worry that I am not doing it right							
3. … if I did HPV self-sampling, I could harm myself							
4. … if I did HPV self-sampling, I could get an infection							
5. … HPV self-sampling is just a way for the government to save money							
6. … if I did HPV self-sampling, I would miss my regular gynecological exam							
7. … I would feel embarrassed doing HPV self-sampling							
8. … I would be willing to pay to do HPV self-sampling over getting the HPV test for free at the doctor's office							

Statement	Strongly disagree (1)	Disagree (2)	Somewhat disagree (3)	Neutral (4)	Somewhat agree (5)	Agree (6)	Strongly agree (7)
I feel that …							
9. … HPV self-sampling would be easy to do							
10. … I would be more comfortable doing the swab by myself using HPV self-sampling than having an HPV test done by a healthcare professional							
11. … to show us that you're reading the questions carefully, please select "somewhat disagree", for this question only [ATTENTION CHECK ITEM]*							
12. … I would prefer doing HPV self-sampling at home because it would save me travelling to see a healthcare professional							
13. … if I did HPV self-sampling, I would be more in control of my body							
14. … if I did HPV self-sampling, I would be concerned about being followed-up properly							

Note: * denotes attention check items used for data cleaning that were not retained in CFA and EFA analyses
