# Supplementary material for: Understanding the Challenges of HPV-Based Cervical Screening: Development and Validation of HPV Testing and Self-Sampling Attitudes and Beliefs Scales
Source: Curr Oncol. 2023 Jan 15;30(1):1206–19. doi: 10.3390/curroncol30010093 (PMC9858121; doi:10.3390/curroncol30010093)
Supplement: Supplementary file 1 [file curroncol-30-00093-s001.zip › Flie S2 Informative Statements.pdf]

Supplementary Material File S2.

Informative Statements

Please carefully read the following information about HPV testing:

Human papillomavirus (HPV) is the most common sexually transmitted infection. Almost all cervical cancers are caused by HPV. Cervical cancer is a highly preventable disease.

The Pap Test

- Cervical cells are looked at in the lab under a microscope to check for abnormal cells
- Currently used for routine cervical cancer screening in Canada
  - Every 2 or 3 years (starting at age 21 or 25, continuing until 65 or 70 depending on the province/territory in which you live)

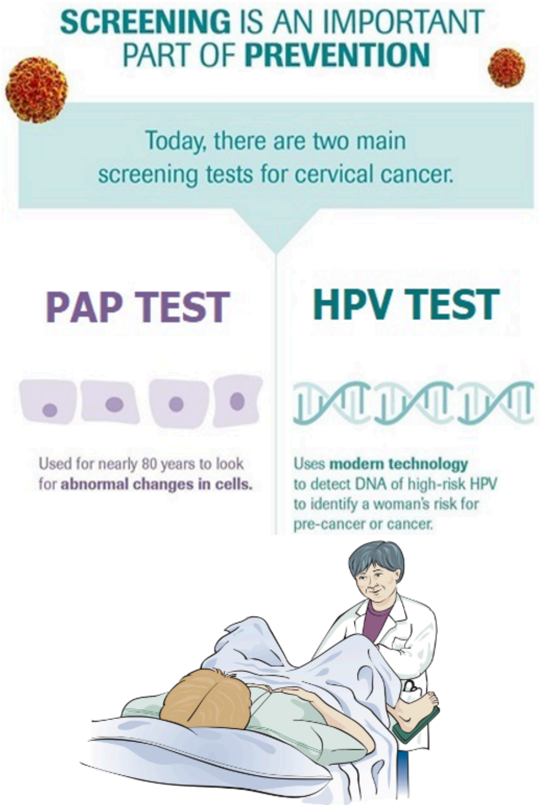

The HPV Test

- Uses specialized technology to look for the presence of HPV DNA
- **Not yet used** for routine cervical cancer screening in Canada
  - Public health authorities are in the process of developing programs to introduce it
- Research shows that if HPV DNA is not found, women are at very low risk for cervical cancer and do not need to screen for cervical cancer as often as with the Pap test (e.g., every 5 years)

For both tests, the procedure to collect the cell sample is the same (see picture above)

Image adapted from CancerCare Manitoba and Roche Diagnostics

Please carefully read the following information about HPV self-sampling:

The HPV test detects the presence of HPV DNA in cervical cells. The collection of the cells for the HPV test can be done by your healthcare professional.

The collection of the cells can also be done by you in privacy. This is called **HPV self-sampling**, where you insert a thin swab into the vagina to collect cervical cells using a kit approved by Health Canada. The sample is then sent to a laboratory for analysis. The results are then returned to your clinic/healthcare provider, who will communicate with you. HPV self-sampling is **not yet used** but is being considered for routine cervical cancer screening in Canada. See the figure containing a description of HPV self-sampling in four steps.

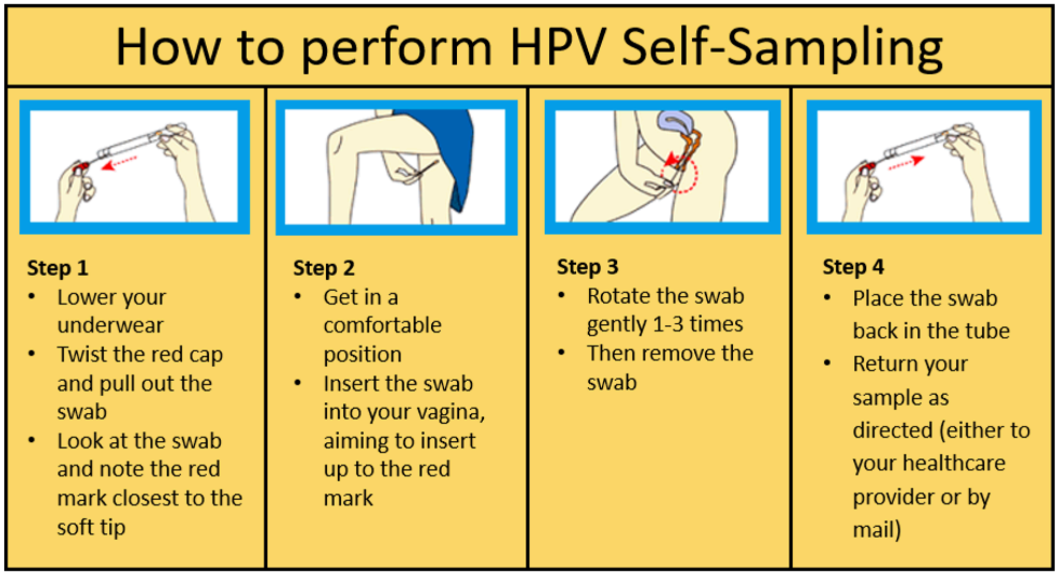

Image adapted from The Royal Australian College of General Practitioners
