# Supplementary material for: Understanding the Challenges of HPV-Based Cervical Screening: Development and Validation of HPV Testing and Self-Sampling Attitudes and Beliefs Scales
Source: Curr Oncol. 2023 Jan 15;30(1):1206–19. doi: 10.3390/curroncol30010093 (PMC9858121; doi:10.3390/curroncol30010093)
Supplement: Supplementary file 1 [file curroncol-30-00093-s001.zip › Flie S3 Exploratory Factor Analyses.pdf]

Supplementary Material File S3.

Exploratory Factor Analyses

Table SA. Parallel analysis HPV test attitudes and beliefs items

Factor	Eigenvalues from dataset n=512	Randomly generated Eigenvalues
1	**7.939**	1.674
2	**5.185**	1.594
3	**2.170**	1.540
4	**1.795**	1.491
5	**1.578**	1.453
6	1.393	1.413
7	1.322	1.377
8	1.244	1.347
9	1.155	1.317
10	1.050	1.287
11	1.034	1.261

Obs: To randomly generate 95 percentile Eigenvalues in parallel analysis we used following specifications: 500 datasets; 512 cases; 44 variables; and principal component analysis. In boldface are Eigenvalues from the dataset that are higher than randomly generated Eigenvalues using parallel analysis suggesting a maximum 5-factor solution.

Table SB. Exploratory Factor Analysis (EFA) for HPV test attitudes and beliefs (44 items) (n=512)

Statement	F1	F2	F3	F4
I feel that …				
1. … I would be embarrassed to show my genitals to a healthcare professional during the HPV test	**.686**	-.019	-.018	-.023
5. … I have other priorities more important than having the HPV test	**.638**	-.079	-.135	-.090
7. … I would be uncomfortable with a male health care professional doing my HPV test	**.581**	-.081	.044	-.095
8. … the HPV test would be painful	**.573**	-.007	.043	.032
19. … I would be embarrassed to get tested for HPV because it is a sexually transmitted infection	**.564**	.198	-.025	.074
12. … I would not need to have the HPV test because I do not have symptoms	**.549**	.113	-.134	-.096
2. … going to see a healthcare professional to have the HPV test would take too much time	**.536**	.028	-.169	.100
11. … I would not need to have the HPV test because I only have one sexual partner	**.498**	.072	-.097	-.046
3. … healthcare professionals doing the HPV test would be rude to me	**.451**	.188	-.263	.120
20. … if the HPV test showed I have HPV, I would be ashamed	**.418**	.171	-.019	.180
33. … I do not have enough information to make a decision about getting screened with the HPV test	**.417**	.122	.075	-.002
4. … that I would not remember to have the HPV test regularly	**.413**	-.051	.072	.052
21. … I would not trust the HPV test as much as the Pap test for my routine cervical cancer screening*	**.365**	.015	-.226	.166
24. … if the HPV test showed I have HPV, I would suspect my partner of being unfaithful	**.227**	.116	.035	.123
41. … my family's opinion about getting the HPV test would be important to me	.101	**.687**	-.020	-.067

40. … my friends' opinion about getting the HPV test would be important to me	.122	**.686**	-.077	-.097
44. … opinions I see on social media (for example, on Facebook, Twitter, Instagram, etc.) about getting the HPV test would be important to me	-.040	**.685**	-.146	.056
42. … my partner's opinion about getting the HPV test would be important to me	.143	**.581**	.104	-.051
22. … if the HPV test showed I have HPV, I would be worried about my ability to have children	.042	**.409**	-.008	.204
6. … if the HPV test showed I have HPV, it would mean I have cancer*	.233	**.306**	-.031	.125
9. … I would need the HPV test because I am at risk of developing cervical cancer*	-.157	**.275**	.111	.274
10. … having the HPV test would prevent me from getting cervical cancer*	-.132	**.206**	.147	.047
26. … if the HPV test showed I have HPV, it is important to follow up on it	-.065	-.179	**.758**	.005
34. … if I learn that I have an HPV infection, I feel that I would need more information to help me deal with the results	.077	-.059	**.701**	.081
16. … having the HPV test would be a good way to identify problems before they become cancer	-.199	-.073	**.675**	.055
17. … the HPV test would be a good way to detect early abnormal changes in the cervix	-.128	-.026	**.580**	.041
18. … the HPV test would be safe	-.253	-.088	**.554**	.023
43. … my healthcare professional's opinion about getting the HPV test would be important to me	-.134	.181	**.538**	.031
35. … it would be important to get a reminder to go for my cervical cancer screening (for example, with a mailed reminder letter)	-.139	.011	**.483**	.254
30. … getting more information about the HPV test would reduce my fear about this test	.073	.230	**.482**	.040
14. … having the HPV test would be entirely up to me	.154	-.118	**.475**	-.102
31. … getting more information about the HPV test may reduce my fear about increasing time between screenings to more than 3 years	-.017	.224	**.466**	.057
39. … if the HPV test showed I have HPV, it would be serious	.110	.157	**.442**	.043
15. … I do not want to have the HPV test because it would affect my sex life*	.276	.364	**-.422**	.139
45. … public health agencies' opinions about getting the HPV test would be important to me	-.088	.232	**.387**	.079
36. … if the HPV test showed I have HPV, I would be comfortable sharing this information with my partner	-.070	-.099	**.360**	-.034
25. … if the HPV test showed I have HPV, I would not really be worried	.071	.064	**-.358**	.053
13. … it would be simple for me to have access to a healthcare professional to receive the HPV test	-.137	.050	**.341**	-.032
23. … if the HPV test showed I have HPV, I would worry about giving my partner HPV*	.035	.041	**.261**	.195
27. … if the HPV test showed I have HPV, this result should not be delivered over the phone*	.111	.138	**.182**	.024
37. … I would be worried about starting screening for cervical cancer with the HPV test at 25 years old instead of 21 years old	.032	-.086	-.096	**.792**
38. … I would be worried about starting screening for cervical cancer with the HPV test at 30 years old instead of 21 years old	.034	-.137	-.036	**.743**

32. … I would be worried about getting tested with the HPV test less often than every 3 years	-.029	.163	-.008	**.428**
29. … if the government increases the time between screenings to more than 3 years, this would be done solely to reduce costs*	.041	-.046	.179	**.239**

Note: Item 28 was not included in EFA because it was an attention check item. In the columns are displayed factor loadings (F1-F4) from EFA. We used maximum likelihood extraction with oblimin rotation for a 4-factor solution on the first random sample n= 512 observations.* denotes items for which factor cross loadings is > 0.6.

Table SC. Parallel analysis HPV self-sampling items

Factor	Eigenvalues from dataset n=512	Randomly generated Eigenvalues
1	**4.091**	1.334
2	**2.700**	1.251
3	1.062	1.192

Obs: To randomly generate 95 percentile Eigenvalues in parallel analysis we used following specifications: 500 datasets; 512 cases; 13 variables; and principal components. In boldface are Eigenvalues from the dataset that are higher than randomly generated Eigenvalues using parallel analysis suggesting a maximum 2-factor solution.

Table SD. Exploratory Factor Analysis (EFA) for HPV self-sampling (13 items) on the first sample (n=512)

Statement	F1	F2
I feel that …		
3. … if I did HPV self-sampling, I could harm myself	**.796**	-.015
4. … if I did HPV self-sampling, I could get an infection	**.793**	.149
7. … I would feel embarrassed doing HPV self-sampling	**.710**	.057
6. … if I did HPV self-sampling, I would miss my regular gynecological exam	**.542**	.255
9. … HPV self-sampling would be easy to do*	**-.531**	.370
2. … if I did HPV self-sampling, I would worry that I am not doing it right	**.503**	-.264
14. … if I did HPV self-sampling, I would be concerned about being followed-up properly	**.502**	-.083
5. … HPV self-sampling is just a way for the government to save money	**.373**	-.035
10. … I would be more comfortable doing the swab by myself using HPV self-sampling than having an HPV test done by a healthcare professional	-.145	**.825**
12. … I would prefer doing HPV self-sampling at home because it would save me travelling to see a healthcare professional	-.283	**.701**
13. … if I did HPV self-sampling, I would be more in control of my body	-.175	**.636**
1. … I would prefer doing HPV self-sampling because it would avoid my family knowing that I am being screened for cervical cancer	.282	**.568**
8. … I would be willing to pay to do HPV self-sampling over getting the HPV test for free at the doctor's office	.141	**.528**

Note: Item 11 was not included in EFA because it was an attention check item. In columns are displayed factor loadings EFA from using maximum likelihood extraction with oblimin rotation for a 2-factor solution on the first random sample n= 512 observations. * denotes items for which factor cross loadings is > 0.6.
