# Supplementary material for: Understanding the Challenges of HPV-Based Cervical Screening: Development and Validation of HPV Testing and Self-Sampling Attitudes and Beliefs Scales
Source: Curr Oncol. 2023 Jan 15;30(1):1206–19. doi: 10.3390/curroncol30010093 (PMC9858121; doi:10.3390/curroncol30010093)
Supplement: Supplementary file 1 [file curroncol-30-00093-s001.zip › Flie S4 Item Response Theory Analyses.pdf]

Supplementary Material File S4.

Item Response Theory Analyses

Table SA. EFA factor loadings and results of IRT for the HPV test attitudes and beliefs items (n=44), first sample (n=512)

Statement	EFA (n=512)	Discrimination (n=512)	Information (n=512)
I feel that …			
**Factor 1**			
1. … I would be embarrassed to show my genitals to a healthcare professional during the HPV test	.686	2.01	1.28
5. … I have other priorities more important than having the HPV test	.638	1.62	0.83
7. … I would be uncomfortable with a male health care professional doing my HPV test	.581	1.22	0.48
8. … the HPV test would be painful	.573	1.30	0.53
19. … I would be embarrassed to get tested for HPV because it is a sexually transmitted infection	.564	1.93	1.18
12. … I would not need to have the HPV test because I do not have symptoms	.549	1.61	0.82
2. … going to see a healthcare professional to have the HPV test would take too much time	.536	1.84	1.08
11. … I would not need to have the HPV test because I only have one sexual partner	.498	1.43	0.64
3. … healthcare professionals doing the HPV test would be rude to me	.451	1.82	1.05
20. … if the HPV test showed I have HPV, I would be ashamed	.418	1.29	0.53
33. … I do not have enough information to make a decision about getting screened with the HPV test	.417	0.90	0.26
4. … that I would not remember to have the HPV test regularly	.413	0.79	0.20
21. … I would not trust the HPV test as much as the Pap test for my routine cervical cancer screening	.365	1.11	0.38
24. … if the HPV test showed I have HPV, I would suspect my partner of being unfaithful	.227	0.59	0.10
**Factor 2**			
41. … my family's opinion about getting the HPV test would be important to me	.687	2.37	1.78
40. … my friends' opinion about getting the HPV test would be important to me	.686	2.24	1.59
44. … opinions I see on social media (for example, on Facebook, Twitter, Instagram, etc.) about getting the HPV test would be important to me	.685	2.05	1.33
42. … my partner's opinion about getting the HPV test would be important to me	.581	1.70	0.92
22. … if the HPV test showed I have HPV, I would be worried about my ability to have children	.409	1.07	0.36
6. … if the HPV test showed I have HPV, it would mean I have cancer	.306	0.81	0.21
9. … I would need the HPV test because I am at risk of developing cervical cancer	.275	0.52	0.09
10. … having the HPV test would prevent me from getting cervical cancer	.206	0.24	0.02
**Factor 3**			
26. … if the HPV test showed I have HPV, it is important to follow up on it	.758	3.25	2.98
34. … if I learn that I have an HPV infection, I feel that I would need more information to help me deal with the results	.701	1.99	1.25

16. … having the HPV test would be a good way to identify problems before they become cancer	.675	2.71	2.10
17. … the HPV test would be a good way to detect early abnormal changes in the cervix	.580	1.96	1.19
18. … the HPV test would be safe	.554	1.92	1.10
43. … my healthcare professional's opinion about getting the HPV test would be important to me	.538	1.71	0.93
35. … it would be important to get a reminder to go for my cervical cancer screening (for example, with a mailed reminder letter)	.483	1.72	0.94
30. … getting more information about the HPV test would reduce my fear about this test	.482	1.10	0.38
14. … having the HPV test would be entirely up to me	.475	0.96	0.28
31. … getting more information about the HPV test may reduce my fear about increasing time between screenings to more than 3 years	.466	1.17	0.43
39. … if the HPV test showed I have HPV, it would be serious	.442	0.97	0.29
15. … I do not want to have the HPV test because it would affect my sex life	-.422	-1.38	0.60
45. … public health agencies' opinions about getting the HPV test would be important to me	.387	1.24	0.48
36. … if the HPV test showed I have HPV, I would be comfortable sharing this information with my partner	.360	1.07	0.36
25. … if the HPV test showed I have HPV, I would not really be worried	-.358	-0.99	0.32
13. … it would be simple for me to have access to a healthcare professional to receive the HPV test	.341	1.14	0.42
23. … if the HPV test showed I have HPV, I would worry about giving my partner HPV	.261	0.81	0.21
27. … if the HPV test showed I have HPV, this result should not be delivered over the phone	.182	0.25	0.02
**Factor 4**			
37. … I would be worried about starting screening for cervical cancer with the HPV test at 25 years old instead of 21 years old	.792	3.26	3.28
38. … I would be worried about starting screening for cervical cancer with the HPV test at 30 years old instead of 21 years old	.743	2.12	1.40
32. … I would be worried about getting tested with the HPV test less often than every 3 years	.428	0.95	0.29
29. … if the government increases the time between screenings to more than 3 years, this would be done solely to reduce costs	.239	0.46	0.07

Note: Highlighted items were retained in the final scale based on results of IRT analyses. In the information column are provided maximum information values as per item information functions.

Table SB. EFA factor loadings and results of IRT for the HPV self-sampling attitudes and beliefs items (n=13), first sample (n=512)

Statement	EFA (n=512)	Discrimination (n=512)	Information (n=512)
I feel that …			
**Factor 1**			
3. … if I did HPV self-sampling, I could harm myself	.796	3.04	2.80
4. … if I did HPV self-sampling, I could get an infection	.793	2.50	1.91
7. … I would feel embarrassed doing HPV self-sampling	.710	2.16	1.46
6. … if I did HPV self-sampling, I would miss my regular gynecological exam	.542	1.24	0.48
9. … HPV self-sampling would be easy to do	-.531	-1.57	0.78
2. … if I did HPV self-sampling, I would worry that I am not doing it right	.503	1.18	0.45
14. … if I did HPV self-sampling, I would be concerned about being followed-up properly	.502	1.19	0.45
5. … HPV self-sampling is just a way for the government to save money	.373	0.88	0.25
**Factor 2**			
10. … I would be more comfortable doing the swab by myself using HPV self-sampling than having an HPV test done by a healthcare professional	.825	4.37	5.80
12. … I would prefer doing HPV self-sampling at home because it would save me travelling to see a healthcare professional	.701	2.34	1.72
13. … if I did HPV self-sampling, I would be more in control of my body	.636	1.99	1.25
1. … I would prefer doing HPV self-sampling because it would avoid my family knowing that I am being screened for cervical cancer	.568	0.92	0.27
8. … I would be willing to pay to do HPV self-sampling over getting the HPV test for free at the doctor's office	.528	0.97	0.30

Note: Highlighted items were retained in the final scale based on IRT analyses. In the information column are provided maximum information values as per item information functions.
