# Supplementary material for: Understanding the Challenges of HPV-Based Cervical Screening: Development and Validation of HPV Testing and Self-Sampling Attitudes and Beliefs Scales
Source: Curr Oncol. 2023 Jan 15;30(1):1206–19. doi: 10.3390/curroncol30010093 (PMC9858121; doi:10.3390/curroncol30010093)

Supplementary Material File S5.

Item Information Functions and Test Characteristic Curves

HPV test Attitudes and Belief Scale

Figure SA. Item information functions (IIFs) and test characteristic curve (TCC) for **Personal barriers** (N=1027)

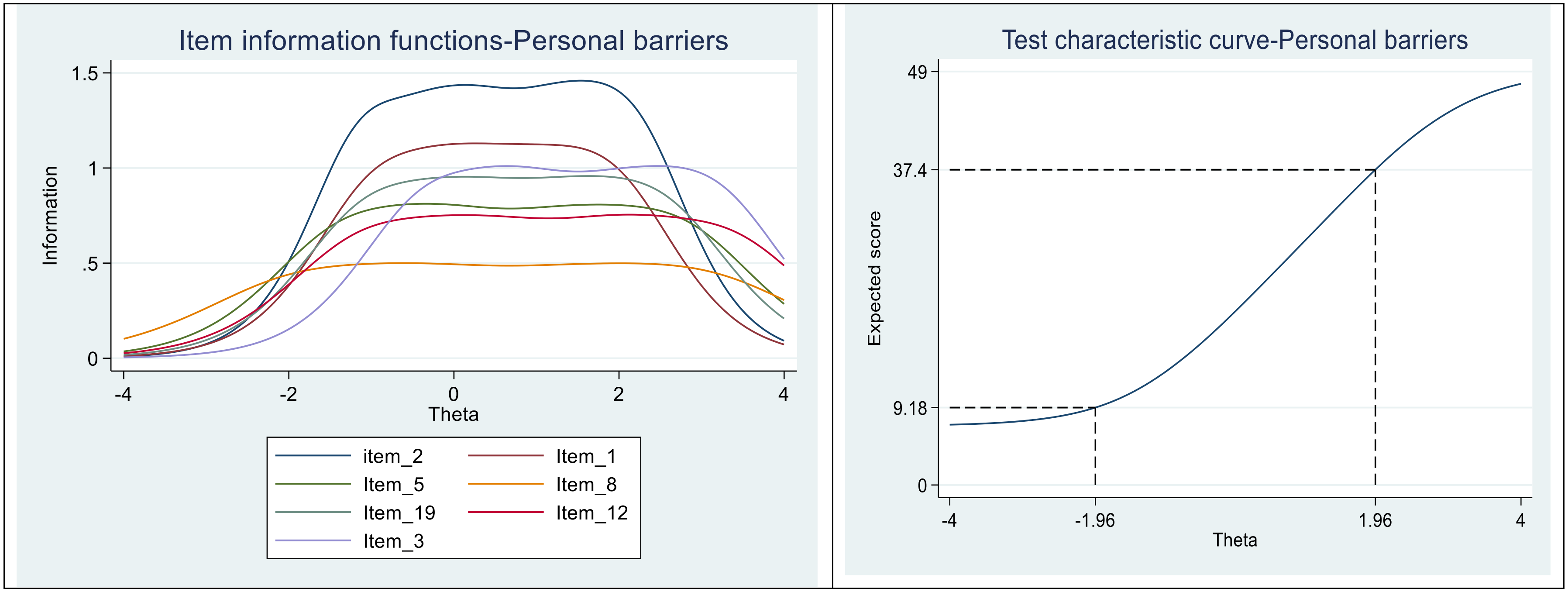

Figure SB. Item information functions (IIFs) and test characteristic curve (TCC) for **<u>Social norms</u>** (N=1027)

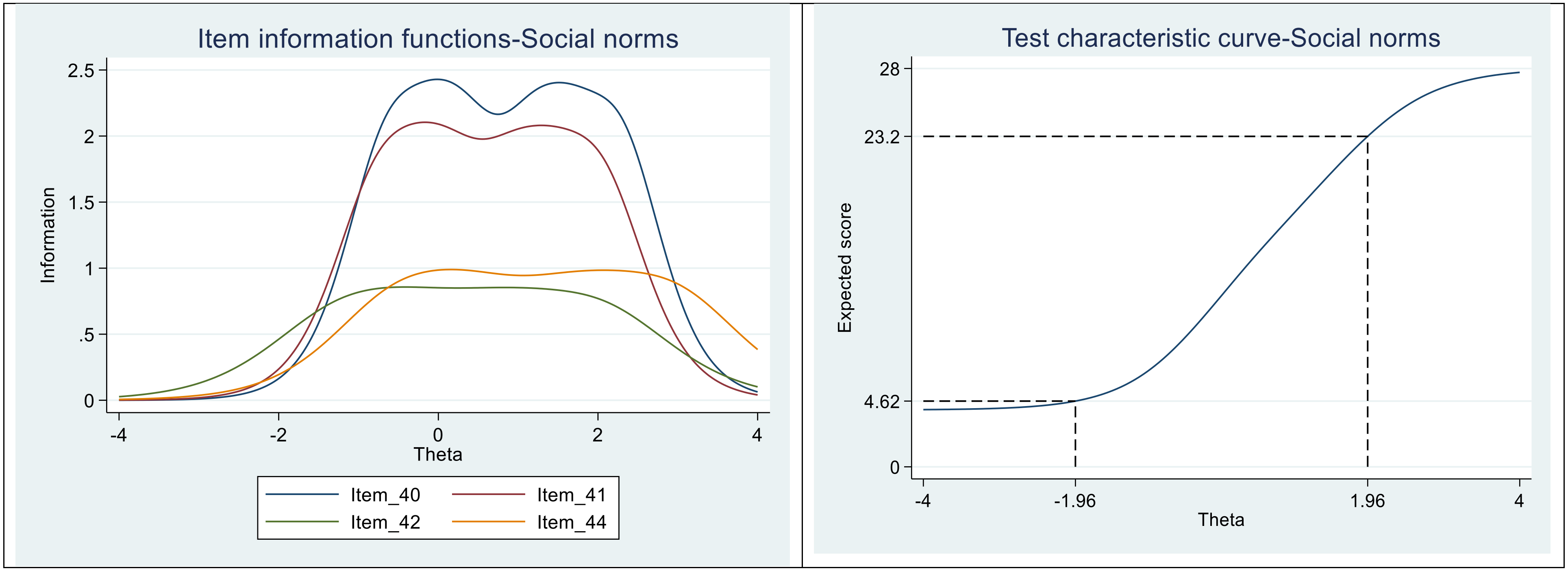

Figure SC. Item information functions (IIFs) and test characteristic curve (TCC) for **Confidence** (N=1027)

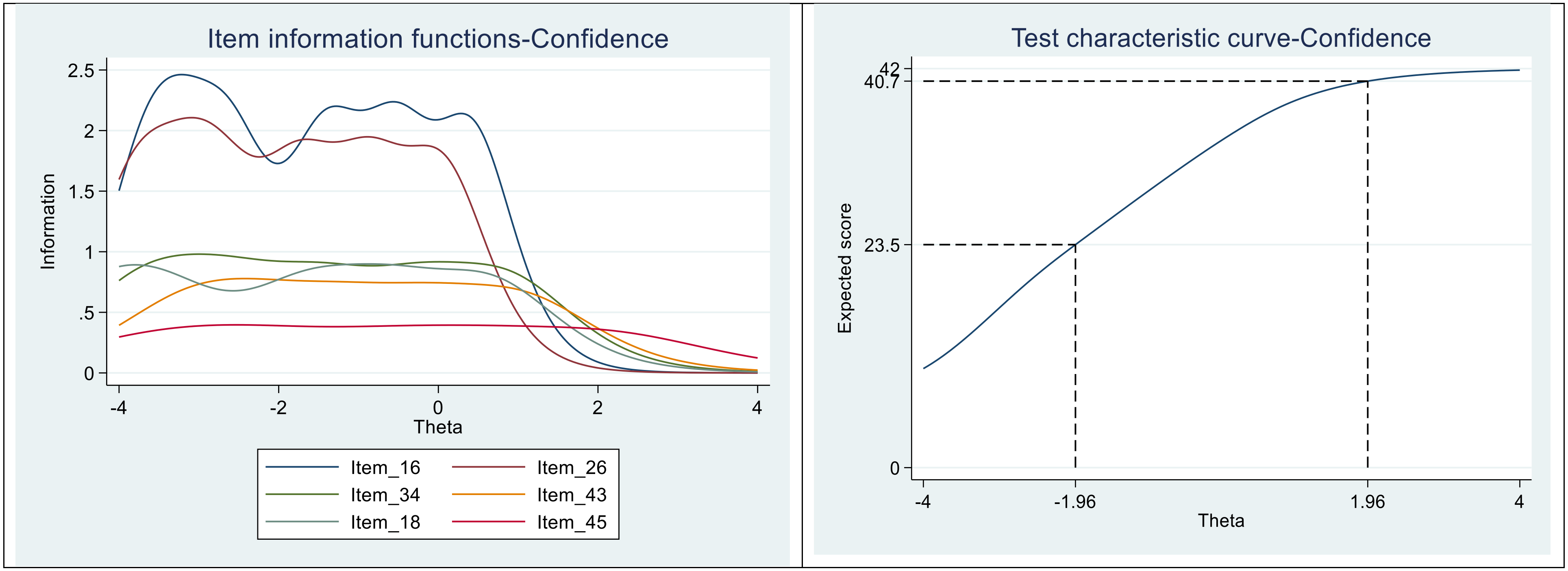

Figure SD. Item information functions (IIFs) and test characteristic curve (TCC) for **worries** (N=1027)

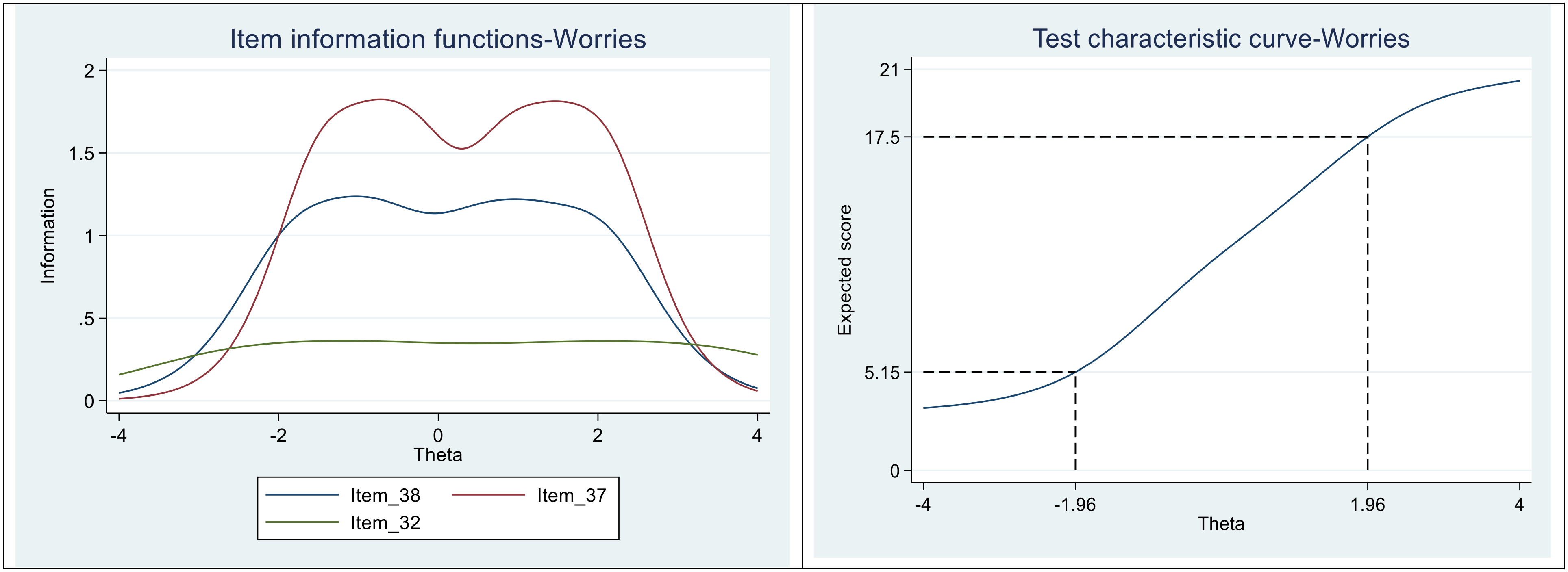

HPV Self-Sampling Attitudes and Belief Scale

Figure SE. Item information functions (IIFs) and test characteristic curve (TCC) for **Concerns** (N=1027)

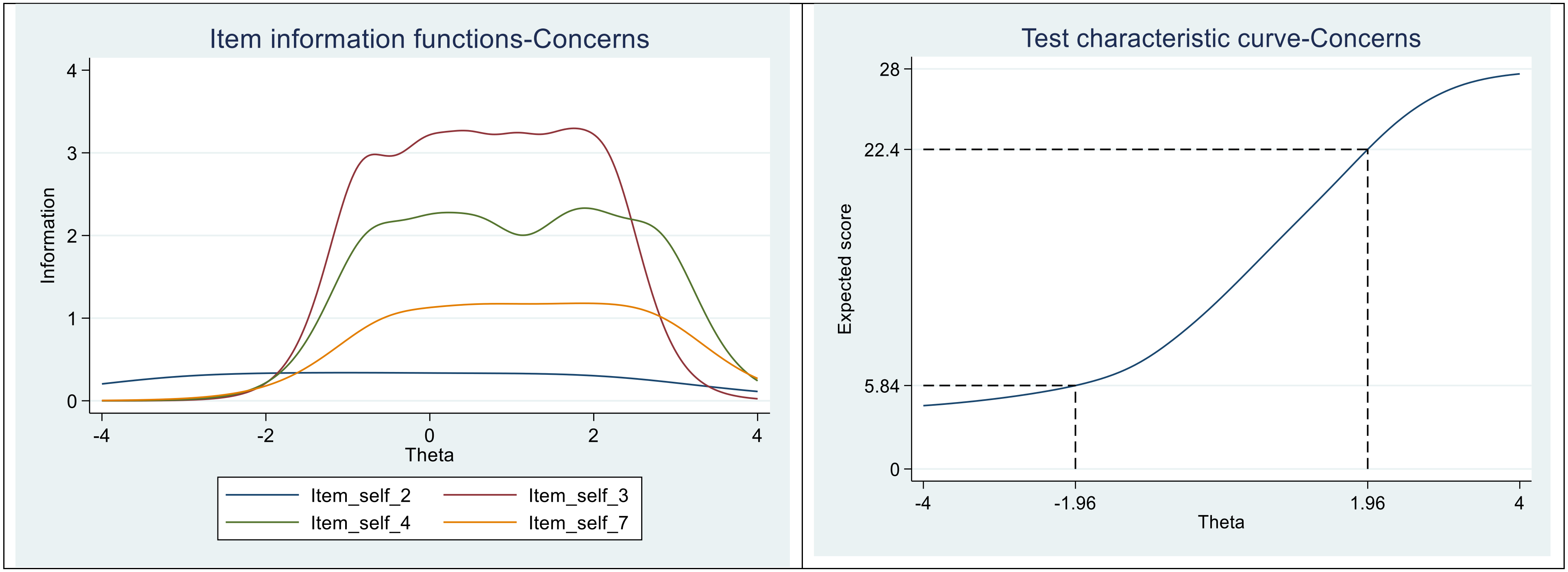

Figure SF. Item information functions (IIFs) and test characteristic curve (TCC) for **Autonomy** (N=1027)

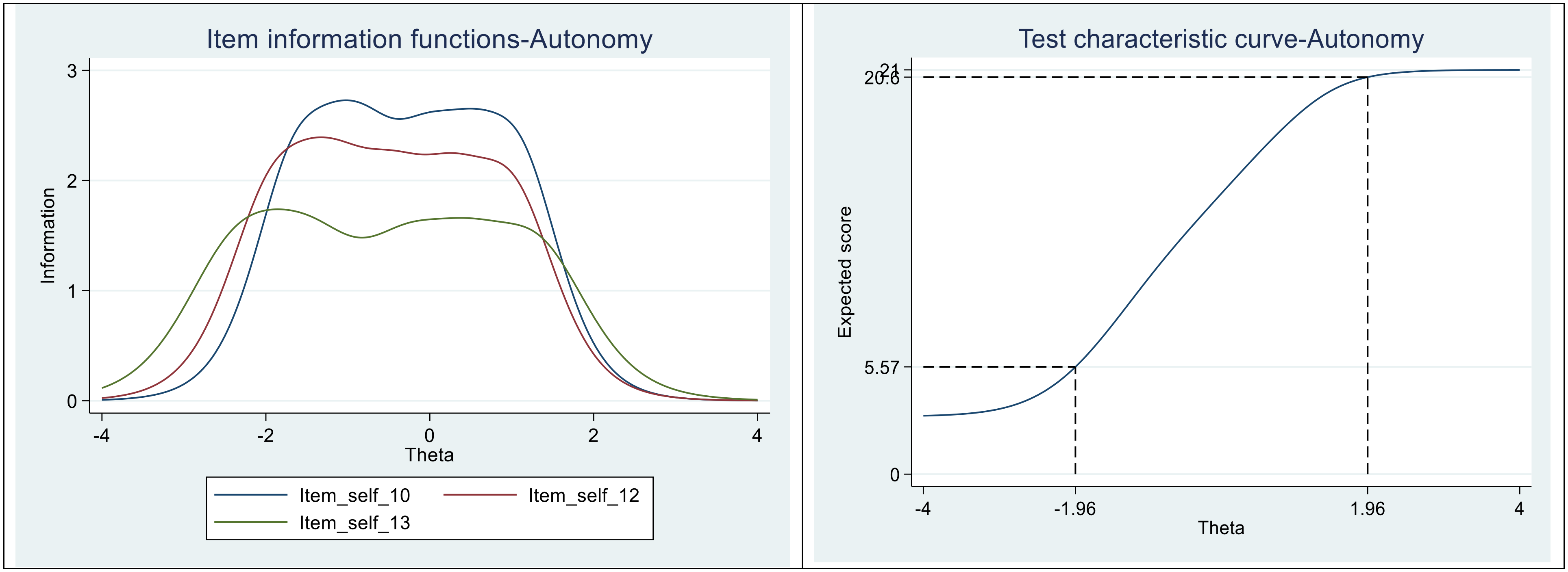

Figure SG. Test information functions (TIF) for items loading on factor 3 (18 items) and retained items (6 items) for the “Confidence” scale (n=512)

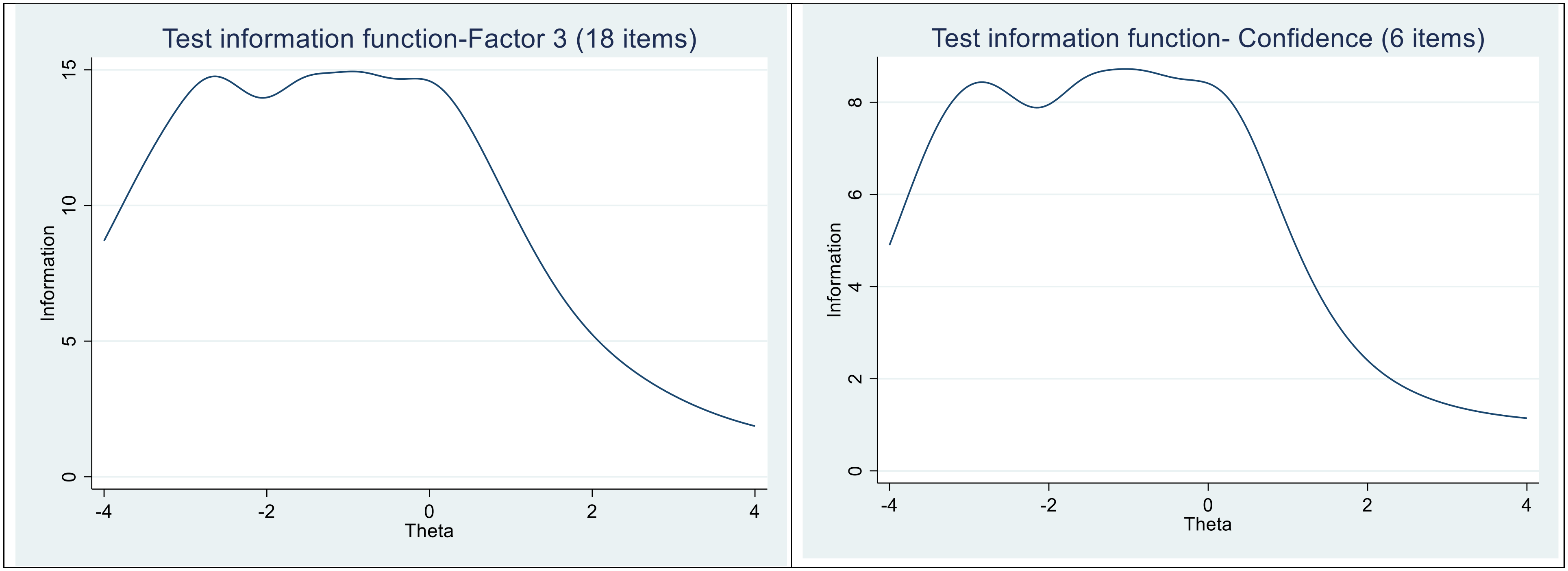

Supplement: Supplementary file 1 [file curroncol-30-00093-s001.zip › Flie S5 Item and Test Characteristic Curves.pdf]
