# Supplementary material for: Understanding the Challenges of HPV-Based Cervical Screening: Development and Validation of HPV Testing and Self-Sampling Attitudes and Beliefs Scales
Source: Curr Oncol. 2023 Jan 15;30(1):1206–19. doi: 10.3390/curroncol30010093 (PMC9858121; doi:10.3390/curroncol30010093)
Supplement: Supplementary file 1 [file curroncol-30-00093-s001.zip › Flie S6 Final Scales.pdf]

Supplementary Material File S6.

Final HPV Attitudes and Beliefs Scale and HPV Self-Sampling Attitudes and Beliefs Scale

Attitudes and Beliefs about HPV Testing (EN)

The following sections will present a series of statements about HPV testing. Please note that we are not asking about your knowledge. Whether or not you have had an HPV test in the past, we wish to know about your attitudes and beliefs. If you are unsure how to answer any question, simply select the answer that best reflects your attitudes and beliefs.

Respond to each prompt with *Strongly disagree (1), Disagree (2), Somewhat disagree (3), Neutral (4), Somewhat agree (5), Agree (6), Strongly agree (7)*

I feel that …

**Factor 1: Personal Barriers**
1. … I would be embarrassed to show my genitals to a healthcare professional during the HPV test
2. … going to see a healthcare professional to have the HPV test would take too much time
3. … healthcare professionals doing the HPV test would be rude to me
4. … I have other priorities more important than having the HPV test
5. … the HPV test would be painful
6. … I would not need to have the HPV test because I do not have symptoms
7. … I would be embarrassed to get tested for HPV because it is a sexually transmitted infection
**Factor 2: Social Norms**
8. … my friends' opinion about getting the HPV test would be important to me
9. … my family's opinion about getting the HPV test would be important to me
10. … my partner's opinion about getting the HPV test would be important to me
11. … opinions I see on social media (for example, on Facebook, Twitter, Instagram, etc.) about getting the HPV test would be important to me
**Factor 3: Confidence**
12. … having the HPV test would be a good way to identify problems before they become cancer
13. … the HPV test would be safe
14. … if the HPV test showed I have HPV, it is important to follow up on it
15. … if I learn that I have an HPV infection, I feel that I would need more information to help me deal with the results
16. … my healthcare professional's opinion about getting the HPV test would be important to me
17. … public health agencies' opinions about getting the HPV test would be important to me
**Factor 4: Worries**
18. … I would be worried about getting tested with the HPV test less often than every 3 years
19. … I would be worried about starting screening for cervical cancer with the HPV test at 25 years old instead of 21 years old
20. … I would be worried about starting screening for cervical cancer with the HPV test at 30 years old instead of 21 years old

Attitudes et opinions relatives au test du VPH (FR)

Les sections suivantes présentent une série d'énoncés sur le test du VPH. Veuillez noter que nous n'évaluons pas vos connaissances. Que vous ayez fait ou non le test du VPH par le passé, nous aimerions connaître votre attitudes et opinions. Si vous ne savez pas comment répondre à l'une de ces questions, veuillez simplement sélectionner la réponse qui reflète le plus votre attitudes et opinions.

Répondez à chaque question par *Je ne suis pas du tout d'accord (1), Je ne suis pas d'accord (2), Je ne suis pas vraiment d'accord (3), Neutre (4), Je suis plutôt d'accord (5), Je suis d'accord (6), Je suis entièrement d'accord (7)*

Je pense que…

**Facteur 1: les barrières personnelles**
1. … cela m'embarrasserait de montrer mes parties génitales à un professionnel de la santé lors d'un test du VPH
2. … consulter un professionnel de la santé pour faire le test du VPH prendrait trop de temps
3. … les professionnels de la santé qui font le test du VPH seraient désagréables avec moi
4. … j'ai d'autres priorités plus importantes que de faire le test du VPH
5. … le test du VPH serait douloureux
6. … je n'aurais pas besoin de faire le test du VPH car je n'ai aucun symptôme
7. … cela m'embarrasserait de faire le test du VPH, car le VPH est une infection transmissible sexuellement
**Facteur 2: les normes sociales**
8. … l'opinion de mes amis sur le fait de réaliser le test du VPH serait importante pour moi
9. … l'opinion de ma famille sur le fait de réaliser le test du VPH serait importante pour moi
10. … l'opinion de mon partenaire sur le fait de réaliser le test du VPH serait importante pour moi
11. … opinions I see on social media (for example, on Facebook, Twitter, Instagram, etc.) about getting the HPV test would be important to me
**Facteur 3: confiance**
12. … faire le test du VPH serait un bon moyen d'identifier un problème avant que cela ne se transforme en cancer
13. … le test du VPH est sécuritaire
14. … si le test du VPH révélait que j'ai le VPH, il serait important que je fasse un suivi approfondi
15. … si j'apprenais que j'ai une infection au VPH, j'aurais besoin de plus d'informations pour m'aider à comprendre ce que signifie ce résultat
16. … l'opinion de mon professionnel de la santé sur le fait de réaliser le test du VPH serait importante pour moi
17. … l'opinion des agences de santé publique sur le fait de réaliser le test du VPH serait importante pour moi
**Facteur 4: les soucis**
18. … cela m'inquiéterait de faire le test du VPH moins souvent qu'à tous les 3 ans
19. … cela m'inquiéterait de commencer le dépistage du cancer du col de l'utérus avec un test du VPH à 25 ans au lieu de 21 ans
20. … cela m'inquiéterait de commencer le dépistage du cancer du col de l'utérus avec un test du VPH à 30 ans au lieu de 21 ans

Attitudes and Beliefs about HPV Self-sampling (EN)

The following sections will present a series of statements about HPV self-sampling. Please note that we are not asking about your knowledge. Whether or not you have done HPV self-sampling in the past, we wish to know your attitudes and beliefs. If you are unsure how to answer any question, simply select the answer that best reflects your attitudes and beliefs.

Respond to each prompt with *Strongly disagree (1), Disagree (2), Somewhat disagree (3), Neutral (4), Somewhat agree (5), Agree (6), Strongly agree (7)*

I feel that…

**Factor 1: Concerns**
1. … if I did HPV self-sampling, I would worry that I am not doing it right
2. … if I did HPV self-sampling, I could harm myself
3. … if I did HPV self-sampling, I could get an infection
4. … I would feel embarrassed doing HPV self-sampling
**Factor 2: Autonomy**
5. … I would be more comfortable doing the swab by myself using HPV self-sampling than having an HPV test done by a healthcare professional
6. … I would prefer doing HPV self-sampling at home because it would save me travelling to see a healthcare professional
7. … if I did HPV self-sampling, I would be more in control of my body

Attitudes et opinions relatives à l'auto-prélèvement du test du VPH (FR)

Les sections suivantes présentent une série d'énoncés sur l'auto-prélèvement du test du VPH. Veuillez noter que nous n'évaluons pas vos connaissances. Que vous ayez réalisé ou non l'auto-prélèvement du test du VPH par le passé, nous aimerions connaître votre attitudes et opinions. Si vous ne savez pas comment répondre à l'une de ces questions, veuillez simplement sélectionner la réponse qui reflète le plus votre attitudes et opinions.

Répondez à chaque question par *Je ne suis pas du tout d'accord (1), Je ne suis pas d'accord (2), Je ne suis pas vraiment d'accord (3), Neutre (4), Je suis plutôt d'accord (5), Je suis d'accord (6), Je suis entièrement d'accord (7)*

Je pense que…

**Facteur 1: les préoccupations**
1. … si je faisais l'auto-prélèvement du test du VPH, j'aurais peur de ne pas le faire correctement
2. … si je faisais l'auto- prélèvement du test du VPH, je pourrais me faire mal
3. … si je faisais l'auto- prélèvement du test du VPH, cela pourrait provoquer une infection
4. … je serais mal à l'aise de faire un auto- prélèvement du test du VPH
**Facteur 2: la commodité**
5. … je serais plus à l'aise à l'idée de faire le prélèvement moi-même à l'aide de l'auto-prélèvement du test du VPH plutôt que de faire réaliser le test par un professionnel de la santé
6. … je préférerais faire l'auto- prélèvement du test du VPH à la maison pour ne pas perdre de temps à aller consulter un professionnel de la santé
7. … si je faisais l'auto- prélèvement du test du VPH, je me sentirais plus en contrôle de mon corps
